# Analysis and application of microbiota in fermentation pit muds used for Chinese strong-flavor liquor production

**DOI:** 10.1186/s13568-026-02070-0

**Published:** 2026-05-14

**Authors:** Hong Dong, Qingyi Fu, Qingyang Wan, Bin Li, Wanxiang Zhang, Xunduan Huang, Xingjie Chen, Laoji Yang, Bing Peng, Guopai Xie, Hongwen Yang, Buchang Zhang, Yansheng Wang

**Affiliations:** 1 School of Life Sciences and Medical Engineering, Anhui University, Hefei, 230601 China; 2 Golden Seed Winery Co., Ltd., Fuyang, 236023 China

**Keywords:** Chinese strong-flavor liquor (CSFL), Fermentation pit mud (FPM), Microbial ecosystem, Biomarker, *S. wolfei*, *C. kluyveri*, Artificial FPM

## Abstract

Chinese strong-flavor liquor (CSFL), the primary type of Chinese liquor in terms of both production and sales, is produced through complex fermentation in pits covered with fermentation pit mud (FPM). FPM, which contains complex microbiota, is crucial for the quality of CSFL. Therefore, monitoring the quality of FPM and acquiring high-quality FPM are important for CSFL manufacturers. However, this requires further study because the relationship between the microbiota and FPM quality remains largely unclear. In this study, high- and low-quality FPM samples were selected based on two-year production data, and the microbiota were sequenced with the HiSeq2500 PE250 platform. Compared with low-quality FPMs, high-quality FPMs exhibited more similar microbiota, and a higher abundance of Euryarchaeota and Firmicutes. Furthermore, the relative abundance of five abundant genera including *Methanobacterium*, *Methanosaeta*, *Clostridium*, *Syntrophomonas* and *Aminobacterium*, were positively correlated with the quality of FPM and could construct a synergistic network helpful for inhabiting FPM and improving the quality of CSFL. At the species level, the abundance of *Clostridium kluyveri* and the new biomarker *Syntrophomonas wolfei* increased in high-quality FPMs and could be rapidly detected using polymerase chain reaction (PCR). By applying functional microbes and knowledge of the microbial ecosystem, artificial FPM was successfully incubated within 50 days, and the yield of high-quality CSFL had an increase of 43.72% compared to that produced with mature FPM. This study provides new insights into further understanding of the microbial ecosystem of FPM and will be useful for producing artificial FPM, as well as for monitoring and improving the quality of FPM used for CSFL production.

**Graphical abstract:**

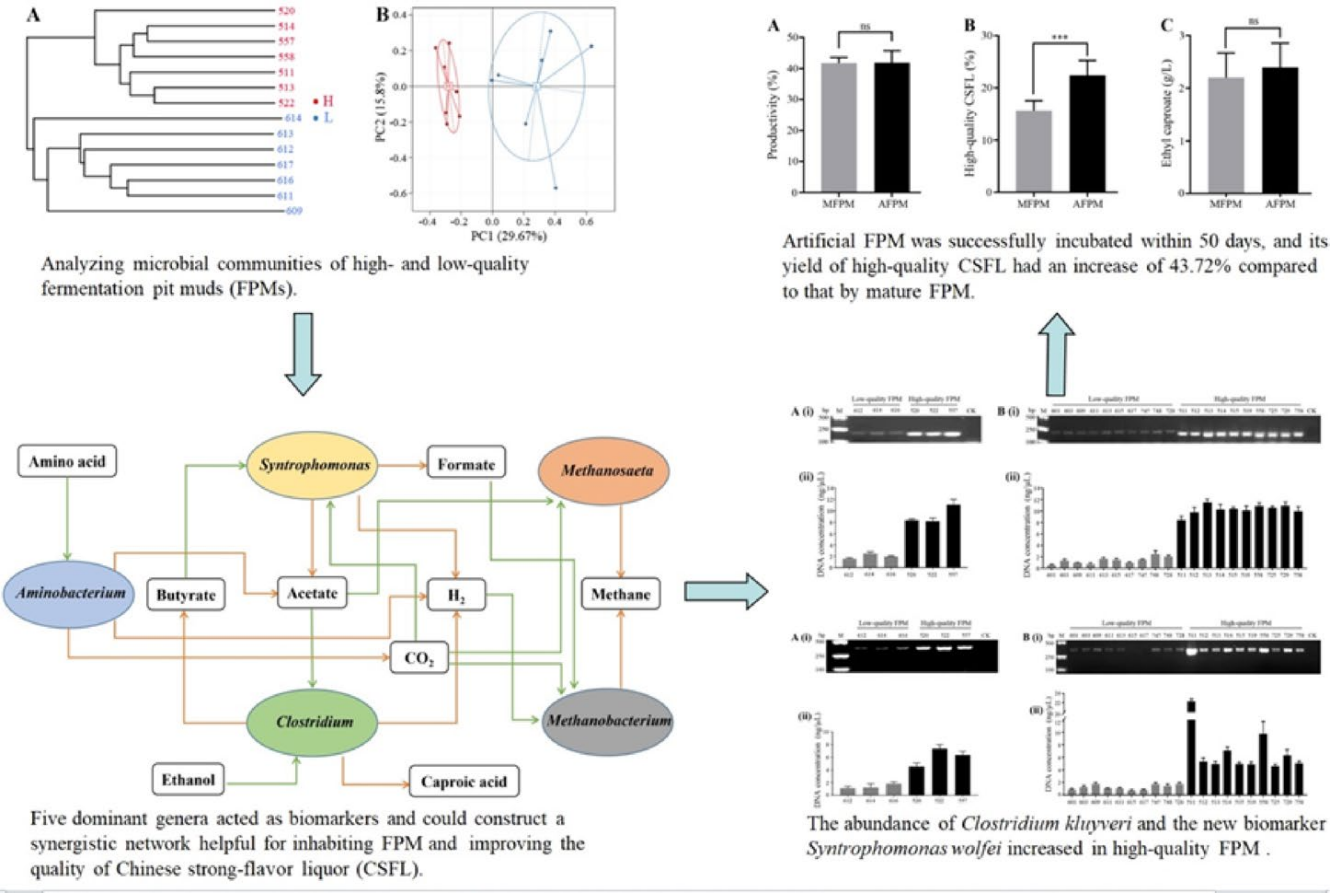

**Supplementary Information:**

The online version contains supplementary material available at 10.1186/s13568-026-02070-0.

## Introduction

Chinese liquor, also known as Chinese Baijiu, is a traditional beverage with an annual yield of millions of tons (Wu et al. 2015; Zou et al. 2018). Chinese strong-flavor liquor (CSFL), also known as Luzhou-flavor liquor, is the primary type of Chinese liquor in terms of both production and sales (Hu et al. 2015; Zou et al. 2018). Fermentation of grains for producing CSFL is carried out in a pit covered with fermentation pit mud (FPM) (Feng et al. 2026).

Generally, FPM, which contains complex microbiota, is a key factor and indispensable for the fermentation of CSFL (Chen et al. 2024; Li et al. 2024; Mei et al. 2025; Zheng et al. 2013). For example, *Clostridium kluyveri*, inhabiting FPM with other microorganisms, produces caproic acid which can be converted to the key CSFL flavor substance ethyl caproate (Hu et al. 2015; Wang et al. 2018). Unfortunately, FPM may be degraded during long-term manipulations (Liu et al. 2022; Sun et al. 2017; Zhang 2010). Monitoring the quality of FPM timely is vital for CSFL production to avoid poor-quality products.

The quality of FPM can be determined based on the CSFL products. However, it takes several months to produce CSFL, by which time it is too late to avoid poor-quality products. Therefore, the quality of FPM is usually determined by sense according to experience, including the characteristics of color, odor, and feel by hand. For example, high-quality FPM often exhibits a gray-black appearance, a strong ester aroma, hydrogen sulfide and ammonia odors, and a moist, soft, and uniform texture (Hu et al. 2016). However, this quality assessment requires super experience and is still subjective. Recently, many studies have focused on exploring the complex microbiota inhabiting FPM. Significant correlations between prokaryotic communities and FPMs with different ages were observed (Han et al. 2024; Tao et al. 2014). Additionally, prokaryotic communities from degraded, normal and high-quality FPMs exhibited obvious differences and significant cooccurrence patterns (Hu et al. 2016). Therefore, the microbiota inhabiting FPM are related to the quality of FPM, and investigating functional microorganisms would be helpful for monitoring the quality of FPM.

In addition to monitoring the quality of FPM, acquiring high-quality FPM is also important for CSFL manufacturers to replace degraded FPM or construct new pits to expand CSFL production. However, a long period of time (even years) and suitable performance are required for the formation of high-quality FPM using normal practices (Liu et al. 2019; Ren et al. 2025; Tao et al. 2014). Therefore, strategies for quickly producing artificial FPM have been adopted by many CSFL manufacturers when high-quality FPM is required. Although many efforts have been made, acquiring high-quality artificial FPM quickly remains difficult and needs to be optimized, partially due to the knowledge about the microbiota in FPM remains largely unclear.

Therefore, investigating the functional microorganisms inhabiting FPM is helpful for monitoring the quality of FPM and producing high-quality FPM. In this study, we investigated two-year production data to select high- and low-quality FPMs and analyzed their microbiota. Quality-related microbes were analyzed and indicators for monitoring the quality of FPM were detected. Additionally, we successfully applied our knowledge of the microbial ecosystem and functional microbes to manufacture artificial FPM for CSFL production.

## Materials and methods

### Collection of FPM samples

High- and low-quality FPMs were collected from Golden Seed Winery Co., Ltd., a famous CSFL manufacturer located in Fuyang City, Anhui Province, China. These FPMs were collected from 26 cellars, including 13 high- and 13 low-quality cellars. The quality of FPMs were determined by two-year production data (Table S1) and the experience of technologists in the enterprise. For high-quality FPMs, the corresponding products regarding the concentration of ethyl caproate in excellent-grade CSFL was up to 3.5 g/L. Five plots of FPM were collected from the bottom of each cellar, and then mixed and designated as one sample.

### Detection of ethyl caproate

Half a milliliter of the sample was extracted with 4.5 mL ether, and the supernatant was analyzed using gas chromatography (GC), which was carried out on a GCMS-QP 2010 Ultra system (Shimadzu, Japan) equipped with a flame ionization detector (FID) and an automatic sampler. Chromatographic separation was achieved using an Agilent DB-FFAP capillary column (30 m × 0.25 mm, 0.25 μm film thickness). The inlet temperature was set at 240 °C. The oven temperature was programmed to start at 100 °C with a 1 min isothermal hold, followed by a temperature ramp of 10 °C/min to 220 °C and maintained for 2 min. The FID temperature was set at 250 °C. The flow rates of hydrogen, air, and make-up gas were 40, 400, and 30 mL/min, respectively. The split ratio was set to 20:1, and the injection volume was 1 µL for each sample. Ethyl caproate with chromatographic pure was used to construct a standard curve, and the concentration of ethyl caproate in each sample could be calculated based on the standard curve.

### DNA extraction, amplification and sequencing

Genomic DNA of each FPM sample was extracted using the PowerSoil^®^ DNA isolation kit (catalog no. 12888-50; MoBio, USA) according to the manufacturer’s instructions. The V4 hypervariable region of the 16S rDNA was amplified with primer set 515F (5’-GTGCCAGCMGCCGCGGTAA-3’) and 806R (5’-GGACTACHVGGGTWTCTAAT-3’). After confirming the quality, the DNA library was sequenced with the HiSeq2500 PE250 system.

### Data analysis

Before obtaining effective tags, the raw sequencing data were filtered and clean data were extracted. Effective tags were clustered into operational taxonomic units (OTUs) using USEARCH (v7.0.1090) with a 97% identity threshold (Edgar 2013). Representative OTUs were annotated with the Ribosomal Database Project (RDP) Classifier 2.2 and Greengenes database at a confidence level of 0.6 (Cole et al. 2014; DeSantis et al. 2006). Alpha and beta diversities were analyzed with mothur (v1.31.2) and QIIME (v1.80), respectively (Caporaso et al. 2010; Schloss et al. 2009). Principal component analysis (PCA) was performed with the ade4 package in R (v3.1.1), and Venn diagrams were generated with VennDiagram in R (v3.1.1). Adonis analysis was carried out with R (v3.5.1).

### Detection of* C. kluyveri* and *S. wolfei* with polymerase chain reaction (PCR)

Species-specific primer pairs Clo-F(5’-GAGGAGCAAATCTCAAAAACTGC-3’)/Clo-R(5’-CCTCCTTGGTTAGACTACGGACTT-3’) and Syn-F(5’-TTCCGGTCGGGTTTTACAGG-3’)/Syn-R(5’-GCTGCCATCGAGCCTATCTT-3’) were used to detect *C. kluyveri* and *S. wolfei*, respectively (Li 2014; Weimer and Stevenson 2012). DNA extracted from the FPM was diluted 10-fold with distilled water and then used as a template for PCR. The PCR reaction mixture was as follows: 2 µL dNTP mix, 1 µL Taq DNA Polymerase, 2.5 µL buffer, 0.5 µL primer F, 0.5 µL primer R, 1 µL DNA template, and 17.5 µL distilled water. PCR protocols were described as follows: 95 ℃ for 10 min, 30 cycles of 94 ℃ for 1 min, 60 ℃ for 1 min, and 72 ℃ for 1 min, then 72 ℃ for 10 min. PCR products (5 µL) were analyzed by gel electrophoresis with 1% agarose. Gel analysis software was used to quantify the abundance of PCR products according to the manufacturer’s instructions, and the value of the CK was used as the background and subtracted.

### Application in manufacturing artificial FPM used for CSFL production

The solid components used to produce artificial FPM were fresh loess (88%), mature FPM (2.5%), Daqu powder (2.5%), bean cake powder (2.5%), wheat powder (4.5%). After mixing these solid components, *C. kluyveri* broth accounting for 20% and 2% of edible alcohol were added. The artificial FPM was incubated in a mud pit and covered with *C. kluyveri* broth to creat anerobic conditions. Additionally, a plastic film was used to cover the pit to prevent the volatilization of the *C. kluyveri* broth. During the incubation, the surface of the artificial FPM was monitored to ensure that the *C. kluyveri* broth was sufficient for coverage and supplemented when needed. After incubation for 50 days, the artificial FPM was used to cover the new pits to produce CSFL.

### Statistical analysis

Data were analyzed with Student’s t-test and the results were stated as the means ± standard deviation (SD). Statistical significance was set at *P* < 0.05.

### Sequencing data accession number

The sequencing data has been deposited in NCBI-SRA under accession number PRJNA1251794.

## Results

### Prokaryotic community structure were obviously different in low- and high-quality FPMs

In total, 674,567 qualified reads were obtained from all FPM samples, and each sample contained 43,939 − 50,951 tags (Table S2). A total of 2626 OTUs were obtained based on 97% similarity of the 16 S rDNA sequences, and the number of OTUs in each sample ranged from 650 to 1443. Rarefaction analysis indicated that all prokaryotic communities were well represented, as the rarefaction curves approached the saturation plateau (Fig. S1).

**Fig. 1:**
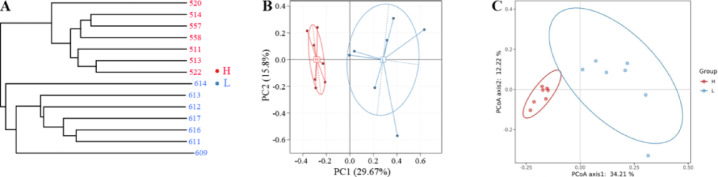
Cluster analysis of microbial communities in high and low-quality FPMs based on bray-curtis distances (**A**), principal component analysis (**B**), and Adonis analysis (**C**). L, low-quality FPM; H, high-quality FPM

All high-quality FPMs clustered together in one group, and low-quality FPMs clustered in another group with more distance (Fig. 1A). Additionally, high-quality FPMs tended to aggregate, whereas low-quality FPMs were relatively dispersed (Fig. 1B and C). These results indicated that high-quality FPMs exhibited obvious differences from low-quality FPMs and contained more similar microbiota.

Although the diversity (Shannon index) showed no significant difference between the two types of FPMs, the microbial richness (observed OTUs and Chao1 index) was obviously higher in the low-quality FPMs (Table 1). Consistently, after excluding 1376 OTUs present in both types of FPMs, high-quality FPMs contained 153 unique OTUs, which were obviously smaller than the 1097 unique OTUs in low-quality FPMs (Fig. S2).

**Table 1:** Microbial community richness and diversity indices for low- and high-quality FPMs

Parameters	Low-quality FPMs	High-quality FPMs
Observed OTUs	1185.00 ± 209.31	834.29 ± 103.82**
Chao1 index	1466.22 ± 179.16	1047.06 ± 146.27**
Shannon index	4.59 ± 0.49	4.20 ± 0.22

** represents *P* < 0.01

Therefore, our research indicates lower richness and more similar microbiota in high-quality FPMs, which is useful for investigating functional microbes and subsequent applications in judging or improving the quality of FPM.

### Relative abundance of predominant microbes were obviously different in low- and high-quality FPMs

For all the samples, 23.29% of the total OTUs were assigned to the archaeal kingdom and 76.71% were affiliated with the bacterial kingdom. In the low-quality FPMs, the dominant phylum of Archaea (> 5% of the total reads) was Euryarchaeota (16.17%), while the dominant phyla of Bacteria were Firmicutes (28.72%), Bacteroidetes (26.30%), and Proteobacteria (12.38%) (Table 2). In the high-quality FPMs, the dominant phylum of Archaea was Euryarchaeota (29.58%), and the dominant phyla of Bacteria were Firmicutes (40.07%) and Bacteroidetes (14.61%). Therefore, Euryarchaeota was the dominant phylum of Archaea in both types of FPMs, and its relative abundance obviously increased in high-quality FPMs. Moreover, as the quality of FPM increased, the relative abundance of Firmicutes increased, whereas that of Bacteroidetes and Proteobacteria declined.

**Table 2:** Relative abundance of dominant phyla (> 5% of the total reads) in low- and high-quality FPMs (%)

Kingdoms	Dominant phyla	Low-quality FPMs	High-quality FPMs
Archaea	Euryarchaeota	16.17 ± 9.20	29.58 ± 6.70**
Bacteria	Firmicutes	28.72 ± 5.89	40.07 ± 11.27*
	Bacteroidetes	26.30 ± 9.99	14.61 ± 5.01*
	Proteobacteria	12.38 ± 10.98	0.57 ± 0.26*

* and ** represent *P* < 0.05 and *P* < 0.01, respectively

Further analysis showed that the relative abundance of six dominant genera (> 1% of the total reads), including *HA73*, *Methanobacterium*, *Methanosaeta*, *Clostridium*, *Syntrophomonas* and *Aminobacterium*, were positively correlated with the quality of FPM (Table 3). These data showed significant differences in microbial abundance in response to the quality of FPM, and useful for investigating functional microbes and subsequent application in judging or improving the quality of FPM.

**Table 3:** Relative abundance of dominant genera (> 1% of the total reads) in low- and high-quality FPMs (%)

Kingdoms	Phyla	Dominant genera	Low-quality FPMs	High-quality FPMs
Archaea	Euryarchaeota	***Methanobacterium***	**4.79 ± 6.15**	**12.98 ± 7.50** ^*^
		*Methanoculleus*	3.09 ± 1.52	4.84 ± 3.29
		***Methanosaeta***	**2.20 ± 2.68**	**6.23 ± 3.61** ^*^
		*Methanosarcina*	3.95 ± 1.86	3.44 ± 1.46
Bacteria	Firmicutes	***Clostridium***	**0.98 ± 0.38**	**2.23 ± 1.17** ^*^
		*Paenisporosarcina*	2.02 ± 1.12	2.73 ± 3.04
		***Proteiniclasticum***	**1.40 ± 1.52**	**0.01 ± 0.01** ^*^
		*Solibacillus*	2.25 ± 2.43	1.18 ± 2.48
		*Sporosarcina*	4.48 ± 5.95	7.03 ± 7.64
		***Syntrophomonas***	**1.08 ± 0.51**	**2.09 ± 0.98** ^*^
	Synergistetes	***Aminobacterium***	**0.75 ± 0.61**	**1.94 ± 1.11** ^*^
		***HA73***	**0.19 ± 0.13**	**1.29 ± 1.21** ^*^
	Proteobacteria	*Acinetobacter*	4.98 ± 9.13	0.02 ± 0.03
		*Pseudomonas*	2.03 ± 4.03	0.00 ± 0.00
	Bacteroidetes	*Ruminofilibacter*	2.00 ± 2.45	1.65 ± 3.26
	Chloroflexi	*T78*	2.04 ± 1.67	1.59 ± 0.44
	Spirochaetes	*Treponema*	2.76 ± 3.03	0.40 ± 0.29

Genera with significant difference in low and high-quality FPMs are bolded.

* represents *P* < 0.05

### The abundance of *S. wolfei* was positively correlated with the quality of FPM

Although sequencing the V4 variable region of the 16 S rDNA could not accurately identify all species of microorganisms, it could provide some data for reference. As shown in Table 4, the relative abundance of *S. wolfei* in high-quality FPMs was higher than that in low-quality FPMs. Considering *Syntrophomonas* is one of the quality-related genera in FPM (Table 3) and *S. wolfei* can utilize butyrate and CO_2_ to produce acetate, H_2_ and formate, which are useful for *Clostridium* and methanogens to grow in FPM (Sieber et al. 2010, 2015), S. *wolfei* may be an important species correlated with the quality of FPM.

**Table 4:** Relative abundance of *S. wolfei* in low- and high-quality FPMs (%)

Low-quality FPMs	Relative abundance of S. wolfei	High-quality FPMs	Relative abundance of S. wolfei
612	0.11	520	0.38
614	0.18	522	1.03
616	0.28	557	0.96
609	0.12	511	1.59
611	0.19	513	0.40
613	0.07	514	0.32
617	0.07	558	0.47
Average	0.15 ± 0.08	Average	0.78 ± 0.50**

** represents *P* < 0.01

**Fig. 2:**
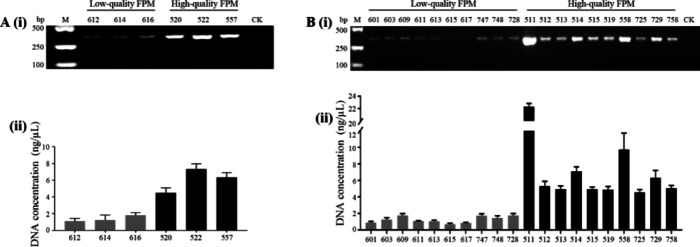
Detection of *S. wolfei* using PCR with specific primers.** A**, Detection of *S. wolfei* in six samples with PCR (i) and the corresponding concentration of target DNA (ii).** B**, Detection of *S. wolfei* in other 20 samples with PCR (i) and the corresponding concentration of target DNA (ii). The PCR products resulted from the samples 612 and 520 were sequenced and identified as *S. wolfei*. M, DNA marker 5000; CK, negative control. The experiment was performed at least three times

The polymerase chain reaction (PCR) is a common and simple method for detecting microorganisms. In the present study, we used this method to detect *S. wolfei*. Using the species-specific primers for *S. wolfei* (Li 2014), we determined the abundance of *S. wolfei* by PCR (Fig. 2). The luminance of the target bands for high-quality FPMs were obviously stronger than that for low-quality FPMs (Fig. 2Ai, Bi). Consistently, the luminance were calculated, and the results showed a higher abundance of *S. wolfei* in high-quality FPMs (Fig. 2Aii, Bii).

Therefore, the abundance of *S. wolfei* was positively correlated with the quality of FPM. Detection and application of *S. wolfei* may be helpful for monitoring and improving the quality of FPM, respectively.

### The abundance of *C. kluyveri* was positively correlated with the quality of FPM

According to previous studies, *C. kluyveri* is a functional habitant and positively correlated with the quality of FPM (Hu et al. 2015, 2016; Zhang et al. 2017a). The relative abundance of *C. kluyveri* was not observed in our HiSeq data, possibly due to the short sequence of the V4 region sequenced in this project, which could not accurately identify this species. Therefore, *C. kluyveri* may also be a candidate related to the quality of FPM in this study.

**Fig. 3:**
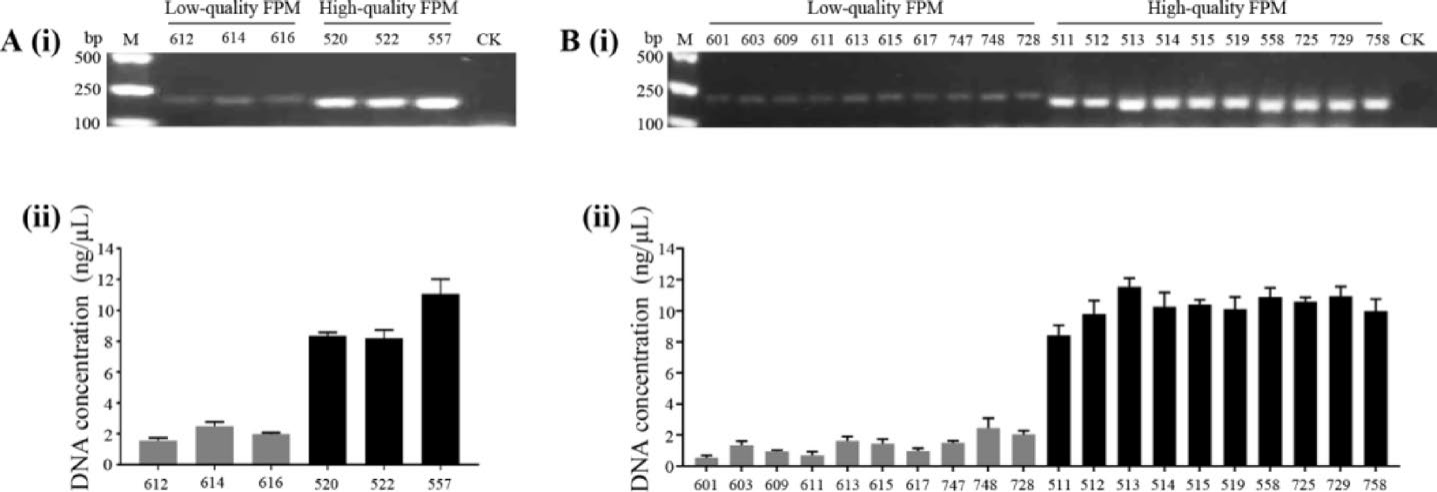
Detection of *C. kluyveri* using PCR with specific primers.** A**, Detection of *C. kluyveri* in six samples with PCR (i) and the corresponding concentration of target DNA (ii).** B**, Detection of *C. kluyveri* in other 20 samples with PCR (i) and the corresponding concentration of target DNA (ii). The PCR products resulted from the samples 612 and 520 were sequenced and identified as *C. kluyveri*. M, DNA marker 5000; CK, negative control. The experiment was performed at least three times

Similar to *S. wolfei*, we investigated the abundance of *C. kluyveri*. Using the species-specific primers for *C. kluyveri* (Weimer and Stevenson 2012), we detected the abundance of *C. kluyveri* by PCR (Fig. 3). The luminance of the target bands for high-quality FPMs were obviously stronger than that for low-quality FPMs, indicating a higher abundance of *C. kluyveri* in high-quality FPMs. Therefore, consistent with previous studies (Hu et al. 2015, 2016; Zhang et al. 2017a), C. *kluyveri* was also positively correlated with the quality of FPM in this study. Detection and application of *C. kluyveri* are also helpful for monitoring and improving the quality of FPM, respectively.

### Application in the manufacture of artificial FPM used for CSFL production

The production of high-quality artificial FPM is crucial for CSFL manufacturers when they need to expand production. However, this is difficult and requires optimization. In this study, based on the investigation of microbiota, we designed a formula for producing artificial FPM by adding functional microbes and their corresponding nutrients. As the source of microbial inoculum, mature FPM was used. Additionally, *C. kluyveri* JZZ (CGMCC No. 11948), isolated from high-quality FPM of Golden Seed Winery Co., Ltd. in our lab in previous work (Peng et al. 2016; Wang et al. 2018), was also supplemented into the artificial FPM. According to previous reports, ethanol and amino acid were useful for *Clostridium* and *Aminobacterium* to grow, respectively (Baena et al. 1998, 2000; Chertkov et al. 2010; Hamdi et al. 2014; Seedorf et al. 2008; Wang et al. 2018). Therefore, to meet the nutrient requirements, ethanol was added, and Daqu powder, bean cake powder and wheat powder were used partially due to their ability to provide amino acids. Based on this formula, we produced artificial FPM within 50 days and used it to cover new pits to produce CSFL.

**Fig. 4:**
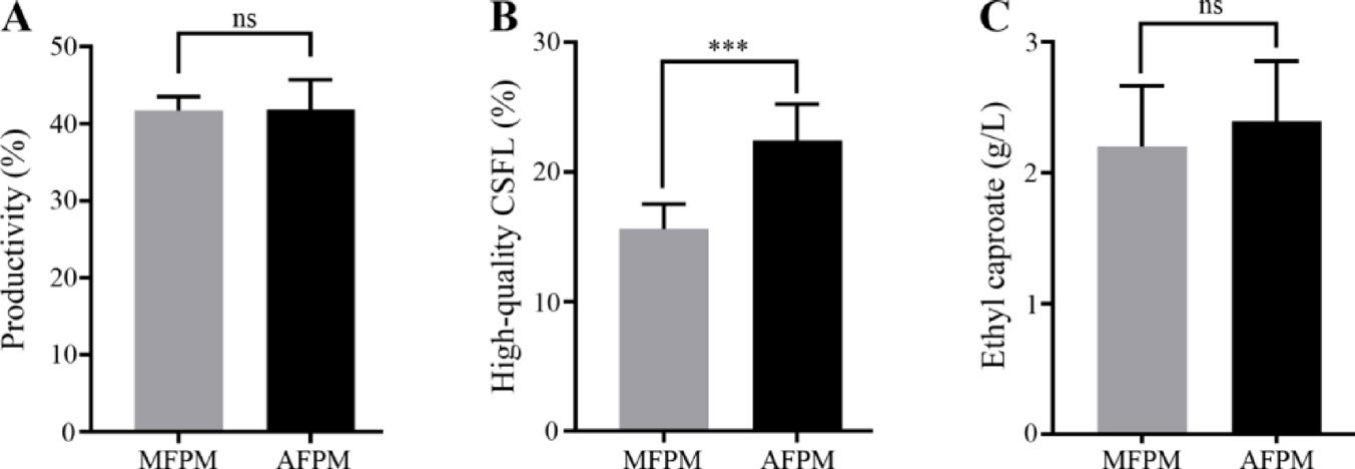
CSFL production by artificial FPM and mature FPM.** A**, Productivity of CSFL;** B**, Proportion of high-quality CSFL in total yield ;** C**, Concentration of ethyl caproate in high-quality CSFL. MFPM, mature FPM; AFPM, artificial FPM. Ten and five pits were used with artificial and mature FPM, respectively. ns, no significant difference; ***, *P* < 0.001

The productivities of CSFL were calculated as yields divided by the quantity of raw material, and the corresponding data resulted by artificial FPM and mature FPM were 41.80% and 41.69%, respectively (Fig. 4A). There was no significant difference in productivity. Importantly, the yield of high-quality CSFL produced by artificial FPM was up to 22.43%, representing an increase of 43.72% than that by mature FPM (Fig. 4B). Meanwhile, the concentration of ethyl caproate in high-quality CSFL produced by artificial FPM was 2.40 g/L, not less than 2.20 g/L produced by mature FPM (Fig. 4C). These results showed successful application in producing artificial FPM in our study, which produced more high-quality CSFL than mature FPM.

## Discussion

Our research indicated lower richness and more similar microbiota in high-quality FPM. These results are consistent with previous reports (Mei et al. 2023; Wang et al. 2020; Zhang et al. 2017b), possibly due to the variation of the microbiota in batch-to-batch fermentation cycles, and ultimately functional microbes were accumulated in high-quality FPM (Gong et al. 2022; Mei et al. 2023). Importantly, the presentation of lower richness and more similar microbiota in high-quality FPM is useful for investigating functional microbes and subsequent application in judging or improving the quality of FPM.

According to previous studies, Euryarchaeota and Firmicutes are regarded as valuable microbes for FPM quality (Fu et al. 2021). For example, the genus *Clostridium*, belonging to the phylum of Firmicutes, is beneficial for the quality of FPM, as *Clostridium* produces caproic acid which can be used to synthesize the key CSFL flavor substance ethyl caproate (Hu et al. 2015; Wang et al. 2018). Meanwhile, the methanogenic bacteria, belonging to the phylum of Euryarchaeota, can grow symbiotically with *Clostridium* and promote the production of caproic acid, which could be converted to ethyl caproate (Han et al. 2024; Hu et al. 2016). Therefore, the increase of Euryarchaeota and Firmicutes in high-quality FPM is reasonable and significant for FPM quality.

**Fig. 5:**
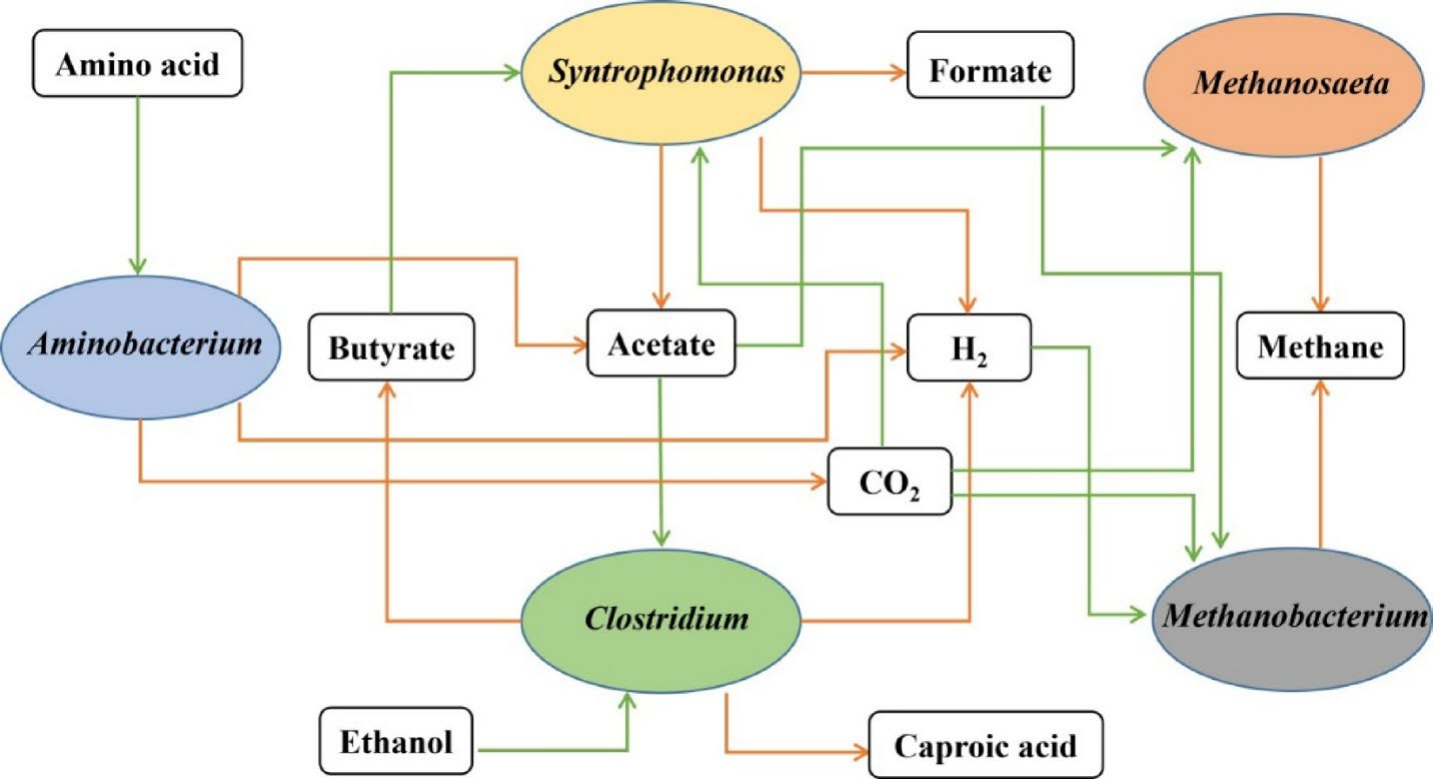
Proposed metabolic network of functional microorganisms. Green and orange arrow represents substrate and production of the specific microorganism, respectively

The relative abundance of six dominant genera (> 1% of the total reads), including *HA73*, *Methanobacterium*, *Methanosaeta*, *Clostridium*, *Syntrophomonas* and *Aminobacterium*, were positively correlated with the quality of FPM (Table 3). Unfortunately, knowledge about *HA73* is limited. Nevertheless, the other five genera, including *Methanobacterium*, *Methanosaeta*, *Clostridium*, *Syntrophomonas* and *Aminobacterium*, could construct a synergistic network for inhabiting FPM according to previous studies (Fig. 5). First, *Aminobacterium* can utilize amino acid to produce acetate, H_2_ and CO_2_ (Baena et al. 1998, 2000; Chertkov et al. 2010; Hamdi et al. 2014). Second, *Clostridium* consumes acetate and ethanol to produce butyrate, caproic acid and H_2_ (Chai et al. 2019; Seedorf et al. 2008; Wang et al. 2018). On the other hand, *Syntrophomonas* consumes butyrate and CO_2_ to produce acetate, H_2_ and formate (Müller et al. 2010; Sieber et al. 2010, 2015). Finally, *Methanosaeta* can use acetate and CO_2_ to produce methane (Mizukami et al. 2006; Smith and Ingram-Smith 2007). Simultaneously, *Methanobacterium* consumes CO_2_, H_2_ and formate to produce methane (Gilmore et al. 2017; Kern et al. 2015; Shcherbakova et al. 2010).

In the aforementioned metabolic network (Fig. 5), the metabolites produced by these microorganisms, including caproic acid, acetate and butyrate, which can be converted to the corresponding esters such as ethyl caproate, are helpful for improving FPM and CSFL quality (Fan and Qian 2006; Feng et al. 2026; Hu et al. 2015). Therefore, the positive correlation between the abundance of these five genera and FPM quality is reasonable, and the utilization of this microbiota ecosystem may be useful for producing artificial FPM or improving the quality of FPM. Additionally, ethanol and amino acid were useful for maintaining the ecosystem (Fig. 5), thus applied in designing the formula for producing artificial FPM in this study.

The abundance of *S. wolfei* was positively correlated with the quality of FPM. To our knowledge, this is the first report of this relationship. According to previous studies, *S. wolfei* can utilize butyrate and CO_2_ to produce acetate, H_2_ and formate (Sieber et al. 2010, 2015). Acetate is a precursor for *Clostridium* to grow and produce caproic acid (Seedorf et al. 2008; Wang et al. 2018), while H_2_ and formate are useful for methanogens to grow (Gilmore et al. 2017; Kern et al. 2015; Shcherbakova et al. 2010). Therefore, *S. wolfei* may be an important species for improving the growth of *Clostridium* and methanogens, which is helpful for improving the quality of FPM (Wang et al. 2018). Consequently, detection and application of *S. wolfei* may be helpful for monitoring and improving the quality of FPM, respectively.

In this study, artificial FPM was successfully incubated within 50 days. Compared with the prolonged natural maturation process (Liu et al. 2019; Ren et al. 2025; Tao et al. 2014), this approach significantly shortened the production time of FPM, demonstrating substantial value for industrial applications. Furthermore, owing to the complexity of FPMs and difficulties in application, previous studies have primarily focused on analysis of FPMs. This study combined the analysis of FPMs and application, which is valuable for CSFL production.

In addition to mature FPM, only *C. kluyveri* was added as a microbial source during the artificial FPM preparation in this study. Future studies can incorporate additional microorganisms to improve the quality of artificial FPM. For instance, microbes such as *S. wolfei* identified in this study, along with other functional microorganisms from the genera *Aminobacterium*, *Methanobacterium* and *Methanosaeta*, can be isolated and utilized for artificial FPM production.

Additionally, the functions of the aforementioned microorganisms discussed in this study were based on existing literatures, and future studies can address their functions in the production of CSFL with valid experiments. Furthermore, because of the limitations of sequencing the V4 region of the 16 S rDNA for species-level resolution, some functional microorganisms may not have been found in this study, and other technologies, such as full-length 16 S rDNA sequencing, may be helpful for improving this problem in future studies.

## Conclusions

In this study, high- and low-quality FPMs were selected, and their microbial ecosystems were investigated using HiSeq technology. The relative abundance of five dominant genera, including *Methanobacterium*, *Methanosaeta*, *Clostridium*, *Syntrophomonas* and *Aminobacterium*, were positively correlated with the quality of FPM and could construct a synergistic network helpful for inhabiting FPM and improving the quality of CSFL. At the species level, the abundance of *C. kluyveri* and the new biomarker *S. wolfei* increased in high-quality FPMs and could be used as indicators for monitoring the quality of FPM. By applying knowledge of the microbial ecosystem and functional microbes, artificial FPM was successfully incubated within 50 days, and the yield of high-quality CSFL had an increase of 43.72% compared with that by mature FPM. This study will be useful for producing artificial FPM, as well as for monitoring and improving the quality of FPM used for CSFL production.

## Supplementary Information

Below is the link to the electronic supplementary material.

Supplementary Material 1

## Data Availability

The datasets used and/or analysed during the current study are available from the corresponding author on reasonable request.
