# Supplementary material for: Analysis and application of microbiota in fermentation pit muds used for Chinese strong-flavor liquor production
Source: AMB Express. 2026 May 14;16:74. doi: 10.1186/s13568-026-02070-0 (PMC13341992; doi:10.1186/s13568-026-02070-0)
Supplement: Supplementary file 1 — Supplementary Material 1 [file 13568_2026_2070_MOESM1_ESM.docx]

**Supplementary Information**

Analysis and application of microbiota in fermentation pit muds used for Chinese strong-flavor liquor production

Hong Dong^a, #^, Qingyi Fu^a, #^, Qingyang Wan^a^, Bin Li^a^, Wanxiang Zhang^a^, Xunduan Huang^a^, Xingjie Chen^b^, Laoji Yang^b^, Bing Peng^b^, Guopai Xie^b^, Hongwen Yang^b^, Buchang Zhang^a^, Yansheng Wang^a,^ *

^a^School of Life Sciences and Medical Engineering, Anhui University, Hefei 230601, China

^b^ Golden Seed Winery Co., Ltd., Fuyang 236023, China

^#^These authors contributed equally to this work.

∗ Corresponding author.

E-mail address: [wangyansheng@ahu.edu.cn](mailto:wangyansheng@ahu.edu.cn)


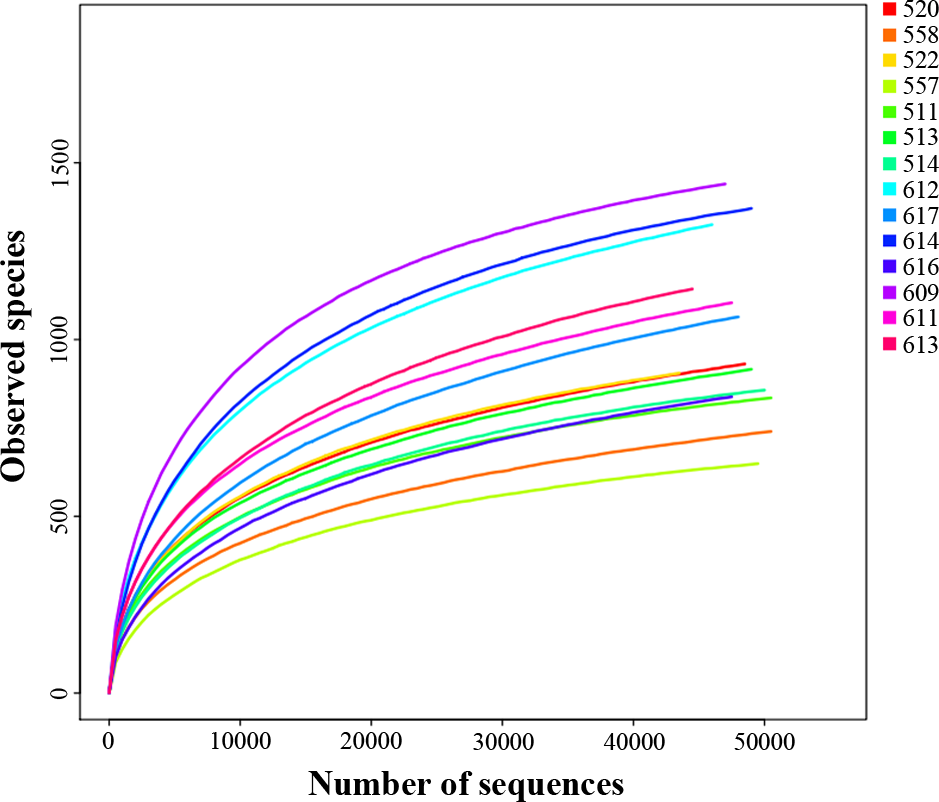


**Fig. S1** Rarefaction curves based on the OTUs at the cutoff of 97% similarity of 16S rDNA sequence.


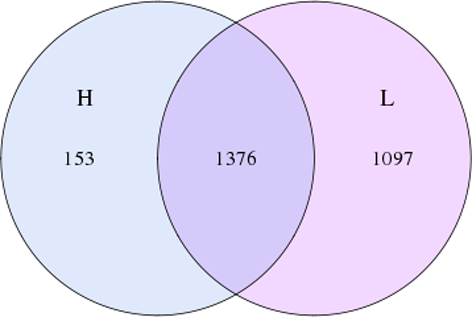


**Fig. S2** Distribution of OTUs in high- and low-quality FPMs.

H, high-quality FPM; L, low-quality FPM.

**Table S1** The concentration of ethyl caproate in excellent-grade CSFL produced by corresponding pit within two years

| **Low-quality FPMs** | **Concentration of**  **ethyl caproate (g/L)** |  | **High-quality**  **FPMs** | **Concentration of**  **ethyl caproate (g/L)** |
| --- | --- | --- | --- | --- |
| **612** | 2.65±0.28 |  | **520** | 4.53±0.74 |
| **614** | 3.21±0.48 |  | **522** | 3.91±0.22 |
| **616** | 2.99±0.21 |  | **557** | 4.12±0.48 |
| **601** | 2.85±0.25 |  | **511** | 3.75±0.23 |
| **603** | 2.99±0.31 |  | **512** | 4.11±0.39 |
| **609** | 3.34±0.36 |  | **513** | 4.30±0.22 |
| **611** | 3.22±0.15 |  | **514** | 4.32±0.44 |
| **613** | 3.25±0.40 |  | **515** | 3.97±0.34 |
| **615** | 2.93±0.56 |  | **519** | 3.84±0.25 |
| **617** | 3.40±0.31 |  | **558** | 3.59±0.24 |
| **747** | 2.98±0.11 |  | **725** | 3.69±0.90 |
| **748** | 2.97±0.15 |  | **729** | 3.66±0.27 |
| **728** | 3.09±0.37 |  | **758** | 3.64±0.28 |

**Table S2** Number of Tags and OTUs.

| **Types of FPMs** | **Sample Name** | **Tag number** | **OTU number** |
| --- | --- | --- | --- |
| Low-quality FPM | 612 | 46074 | 1325 |
|  | 614 | 49245 | 1372 |
|  | 616 | 47649 | 839 |
|  | 609 | 47449 | 1443 |
|  | 611 | 47640 | 1105 |
|  | 613 | 44644 | 1144 |
|  | 617 | 48445 | 1067 |
| High-quality FPM | 520 | 48798 | 932 |
|  | 522 | 43939 | 907 |
|  | 557 | 49830 | 650 |
|  | 511 | 50556 | 835 |
|  | 513 | 49307 | 917 |
|  | 514 | 50040 | 857 |
|  | 558 | 50951 | 742 |
